# Geographic epidemiology of cardiometabolic risk factors in middle class urban residents in India: cross–sectional study

**DOI:** 10.7189/jogh.05.010411

**Published:** 2015-06

**Authors:** Rajeev Gupta, Krishna Kumar Sharma, Bal Kishan Gupta, Arvind Gupta, Banshi Saboo, Anuj Maheshwari, Tulika Mahanta, Prakash C Deedwania

**Affiliations:** 1 Fortis Escorts Hospital, Jaipur, India; 2 SP Medical College and Associated Group of Hospitals, Bikaner, India; 3 Jaipur Diabetes Research Centre, Jaipur, India; 4 Diabetes Care and Hormone Clinic, Ahmedabad, India; 5 BBD College of Dental Sciences, Lucknow, India; 6 Assam Medical College, Dibrugarh, India; 7 University of California San Francisco, Fresno, California, USA

## Abstract

**Objective:**

To determine epidemiology of cardiovascular risk factors according to geographic distribution and macrolevel social development index among urban middle class subjects in India.

**Methods:**

We performed cross-sectional surveys in 11 cities in India during years 2005–2009. 6198 subjects aged 20–75 years (men 3426, women 2772, response 62%) were evaluated for cardiovascular risk factors. Cities were grouped according to geographic distribution into northern (3 cities, n = 1321), western (2 cities, n = 1814), southern (3 cities, n = 1237) and eastern (3 cities, n = 1826). They were also grouped according to human social development index into low (3 cities, n = 1794), middle (5 cities, n = 2634) and high (3 cities, n = 1825). Standard definitions were used to determine risk factors. Differences in risk factors were evaluated using χ^2^ test. Trends were examined by least squares regression.

**Findings:**

Age–adjusted prevalence (95% confidence intervals) of various risk factors was: low physical activity 42.1% (40.9–43.3), high dietary fat 49.9% (47.8–52.0), low fruit/vegetables 26.9% (25.8–28.0), smoking 10.1% (9.1–11.1), smokeless tobacco use 9.8% (9.1–10.5), overweight 42.9% (41.7–44.1), obesity 11.6% (10.8–12.4), high waist circumference 45.5% (44.3–46.7), high waist–hip ratio 75.7% (74.7–76.8), hypertension 31.6% (30.4–32.8), hypercholesterolemia 25.0% (23.9–26.9), low HDL cholesterol 42.5% (41.3–43.7), hypertriglyceridemia 36.9% (35.7–38.1), diabetes 15.7% (14.8–16.6), and metabolic syndrome 35.7% (34.5–36.9). Compared with national average, prevalence of most risk factors was not significantly different in various geographic regions, however, cities in eastern region had significantly lower prevalence of overweight, hypertension, hypercholesterolemia, diabetes and metabolic syndrome compared with other regions (*P* < 0.05 for various comparisons). It was also observed that cities with low human social development index had lowest prevalence of these risk factors in both sexes (*P* < 0.05).

**Conclusions:**

Urban middle–class men and women in eastern region of India have significantly lower cardiometabolic risk factors compared to northern, western and southern regions. Low human social development index cities have lower risk factor prevalence.

Cardiovascular diseases are one of the most important causes of morbidity and mortality in low and middle income countries, including India [1]. However, there are substantial within–country variations in cardiovascular morbidity and studies in Europe and North America have reported substantial national, urban–rural and regional variations in cardiovascular disease incidence and mortality [2,3]. These differences are due to variations in lifestyles (dietary factors, physical activity and smoking), biological risk factors (hypertension, lipid levels, diabetes and metabolic syndrome) and social factors [1]. Macrolevel social factors [4] could be important causes of these differences but have not been well studied. Macro–level factors that influence cardiovascular risk are area based measures (urban or rural), measures of living conditions, measures of income inequality, human development index and status of health care delivery [4,5]. Individual–level social factors are social status, education, income, occupation, employment status, lifespan social class, and factors that influence adherence to lifestyles and medical treatment [5].

In India, significant geographic variations in cardiovascular mortality have been reported [6]. Studies have also reported significant urban–rural differences in cardiovascular morbidity and risk factors [7]. Studies which used similar methodology reported greater prevalence of obesity, abdominal obesity, hypertension, hypercholesterolemia and diabetes in urban as compared to rural populations [8-11]. Reviews have reported significant geographic differences in prevalence of smoking [12], obesity [13], hypertension [14], dyslipidemia [15], and diabetes [16] in India. These geographic differences in cardiovascular risk factors could be due to ethnic and sociocultural differences as well as differences in macrolevel socioeconomic factors such as degree of urbanization and human and social development indices [17]. Study of macrolevel factors is important because these are influenced by national and regional policies and quality of local governance [4,5]. Moreover, macrolevel factors are better amenable to social engineering [18]. Social engineering has been defined as a process through which the state can improve education, health care, housing and other basic facilities to improve quality of life and address problems of ill–health, poverty, unemployment and slow development [19].

Urbanization is rapidly increasing in India and this population is poised to increase from the current 400 million (35% of the country) to more than a billion in the next 30 years [20]. There is, therefore, a need to evaluate current prevalence of cardiometabolic risk factors in India, especially urban locations. There is also a need to evaluate risk factors in the urban middle–class which is one of the larger segments of Indian society [21]. It has been predicted that in the next 30 years more than 70% of the national population shall be urban and most would be in the middle class [20,21]. Accordingly, we designed the India Heart Watch study to identify prevalence of cardiometabolic risk factors among middle–class subjects living in urban locations in different regions of India [22]. We defined middle–class subjects as those living in middle–class locations as defined by local municipal councils. Although there could be state–level variations in such definitions, previous reports from India have shown insignificant heterogeneity [23]. To determine macrolevel determinants of cardiovascular risk factors in the urban middle–class, we first evaluated influence of geographic differences after dividing the country into four regions– north, south, east and west with 2–3 cities in each (**Figure 1**). There is a significant socioeconomic heterogeneity in various geographic regions of the country [23,24]. Eastern and central Indian regions as well as some states in northern India (called empowered action groups states) are less developed as compared to western and southern regions [24]. This may vitiate the geographic differences which also include regional and local socioeconomic development [23-25]. Therefore, to better determine macrolevel determinants of cardiovascular risk, we used a novel social development index which is a measure of poverty and its social determinants and is a composite of demography, health care, basic amenities, education, economic deprivation and social deprivation (**Table 1**) [25].

**Figure 1:**
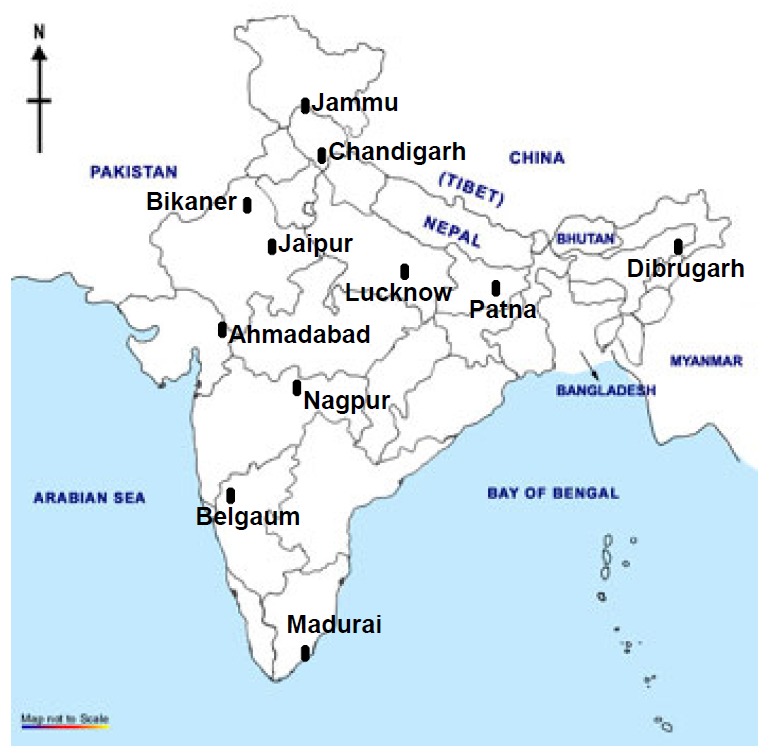
Map of India showing various locations in the India Heart Watch study.

**Table 1:** Indicators, database indicators and database used in estimation of social development index

Demographic indicators	Contraceptive prevalence rate Total fertility rate Infant mortality rate
Health indicators	Percentage of institutional deliver Percentage of undernourished children
Educational attainment indicators	Literacy rate Pupil–teacher ratio School attendance rate
Basic amenities indicators	Percent households which live in *pucca* house Households with access to safe drinking water Households with access to toilet facility Households with electricity connection
Economic deprivation indicators	Household economic date from National Sample Survey 61^st^ round (2004–5) and 62^nd^ round (2005–6)
Social deprivation indicators	Disparity ratio between scheduled castes and general population in literacy rates Disparity ratio between scheduled tribes and general population in literacy rates Male–female disparity ratio in education Female–total unemployment rate ratio Disparity ratio of per capita expenditure of Muslim population with total population Child sex ratio

## METHODS

A multisite study to identify prevalence of cardiovascular risk factors and their socio–demographic determinants was organised among urban subjects in India. Rationale for the study has been reported earlier [22]. Protocol was approved by the institutional ethics committee of the national coordinating centre. Written informed consent was obtained from each participant. The study performa was developed according to recommendations of the World Health Organization (WHO) [26].

### Regions and investigators

We planned the study to identify prevalence of cardiometabolic risk factors and their determinants among urban subjects in India [27]. Medium sized cities were identified in each of the large states of India and investigators who had a track record of research in cardiovascular or diabetes epidemiology were invited to participate. 20 investigators were invited from all large states of India and 11 participated. A steering committee meeting with all the investigators was organised at initiation of the study where the study protocol was discussed and developed. The meeting was followed by training in salient features of questionnaire and techniques of evaluation to ensure uniformity in recruitment and data collection. The cities are in northern (Jammu, Chandigarh, Bikaner), western (Ahmadabad, Jaipur), eastern (Lucknow, Patna, Dibrugarh) and southern (Madurai, Belgaum, Nagpur) regions of India (**Figure 1**). Salient demographic characteristics of these cities are shown in **Table 2**.

**Table 2:** Socio–demographic characteristics of the study sites

Location (City, State)	Population in millions (Census 2011)	Females/1000 Males	Literacy rate (%)	Urban slums (%)	Human Development Index	Social Development Index	Present study sample
**Northern India**							
Jammu (Jammu & Kashmir)	0.51	889	89.6	–	0.529	0.51	320
Chandigarh (Chandigarh)	0.96	818	86.0	13.2	0.784	0.77	502
Bikaner (Rajasthan)	0.65	852	66.0	18.5	0.434	0.38	499
**Western India**							
Ahmedabad (Gujarat)	6.36	897	89.0	13.5	0.577	0.67	490
Jaipur (Rajasthan)	3.05	907	76.4	15.1	0.434	0.51	1324
**Southern India**							
Belgaum (Karnataka)	0.61	969	78.0	11.0	0.519	0.66	50
Nagpur (Maharashtra)	2.50	961	93.1	35.9	0.572	0.73	264
Madurai (Tamilnadu)	1.47	999	81.9	23.8	0.570	0.73	923
**Eastern India**							
Lucknow (Uttar Pradesh)	2.90	923	84.7	8.2	0.380	0.34	835
Patna (Bihar)	1.68	882	84.7	0.3	0.367	0.23	491
Dibrugarh (Assam)	0.15	952	89.5	–	0.444	0.63	500

### Sampling

The study data were collected in the years 2006–2010. Simple cluster sampling was performed at each site. A middle–class location was identified at each city based on municipal classification derived from cost of land, type of housing, public facilities (roads, sanitation, water supply, electricity, gas supply, etc.), and educational and medical facilities as reported earlier [27]. Sample size of about 250 men and 250 women (n = 500) at each site is considered adequate by the WHO to identify 20% difference in mean level of biophysical and biochemical risk factors [26]. The sample size required for 85% chance of recognizing a specified difference in rates (power 1–β) between two populations, significant at 5% level in a two–tailed test, when true prevalence rates are 10% and 5% is 490 subjects. These prevalence rates are similar to previous studies on diabetes prevalence (the lowest prevalent cardiovascular risk factor) from India. For continuous variables this sample size would have 85% chance of recognizing a difference in mean value of 1 with SD of 5 [26]. Accordingly, we invited 800–1000 subjects in each location to ensure participation of at least 500 subjects at each site estimating a response of 70% as reported in previous studies [28]. Sample sizes at some sites was low due to low recruitments (eg, Belgaum) and oversampling was performed at high recruiting sites (eg, Jaipur, Madurai) to have adequate geographic representation (**Table 2**). At each site a uniform protocol of recruitment was followed [27]. The surveys were preceded by meetings with community leaders to ensure good participation. Subjects were invited in fasting state to a community centre of medical centre within each locality either twice or thrice a week depending upon the investigator’s schedule. Inclusion criteria were all adults aged ≥20–75 years living in the particular location. Subjects who were confined to home with severe debilitating disease, those not likely to survive beyond 6 months and pregnant women were excluded.

### Measurements

The study performa was filled by the research worker employed by the site investigator after details were inquired from the subject. Apart from demographic history, details of educational status, history of known hypertension, diabetes, lipid abnormalities and cardiovascular disease were inquired [27]. Details regarding smoking and smokeless tobacco use, alcohol intake, dietary fat and fruits and vegetables intake were assessed as reported previously [29]. Details of physical activity were inquired for exact daily duration (minutes) of work related, commute related and leisure time physical activity [29]. Equipments for measurement of height, weight, waist and hip size and blood pressure were similar to ensure uniformity as suggested by WHO guidelines [26]. Sitting blood pressure was measured after at least 5–minute rest using standardised instruments. Three readings were obtained and were averaged for the data analysis. Fasting blood sample was obtained from all individuals after 8–10 hours fast. Fasting state was determined according to self–reports. The blood samples were obtained at community centres by technicians from an accredited national laboratory– Thyrocare Technologies Ltd, Mumbai, India (www.thyrocare.com). Blood glucose was measured at the local biochemistry facility of these laboratories. Blood for cholesterol, cholesterol lipoproteins and triglycerides estimation was transported under dry ice to the national referral laboratory where all the blood samples were analysed using uniform protocol. Cholesterol, high density lipoprotein (HDL) cholesterol and triglyceride levels were measured using enzyme–based assays with internal and external quality control (www.thyrocare.com) as reported earlier [30]. Values of low density lipoprotein (LDL) cholesterol were calculated using Friedwald’s formula: LDL cholesterol = total cholesterol – HDL cholesterol + triglycerides/5).

### Diagnostic criteria

The cities were grouped into four geographic regions– northern, western, southern and eastern (**Figure 1**). Demographic details of the cities are shown in **Table 2**. Although the population of the cities varied from more than 6 million (Ahmedabad) to less than half a million, they are not very dissimilar in other socio–demographic characteristics such as literacy, housing and human development index (**Table 2**). Cities were also grouped according to urban social development index. Computation of social development index is similar to human development index developed by the United Nations Development Program and uses the range equalization method wherein each indicator is divided by range of the particular indicator so that scale–free values vary between zero and unity (**Table 1**) [25]. Details of its calculation for Indian urban locations are provided in the publication [25]. This index is a measure of poverty and its social and health manifestations (demographic and health indicators) and their social determinants (education, economic deprivation, social deprivation and amenities). The cities were classified according to this index into tertiles of high (Chandigarh 0.77, Madurai 0.73, and Nagpur 0.73), medium (Ahmedabad 0.67, Belgaum 0.66, Dibrugarh 0.63, Jaipur 0.51, Jammu 0.51) and low (Bikaner 0.38, Lucknow 0.34, Patna 0.23) human social development index.

Details of other diagnostic criteria have been reported earlier [27]. Smokers included subjects who smoked cigarettes, *bidis*, or other smoked forms of tobacco daily, past smokers were subjects who had smoked for at least 1 year and had stopped more than a year ago. Users of other forms of tobacco (oral, nasal, etc) were classified as smokeless tobacco use. Subjects consuming ≥20 g visible fat daily were categorized as high fat intake. This corresponds to total fat intake of >40 g/d reported in a previous study from India [31] and corresponds to percent energy intake from fat (fat en%) of >30% [32]. The WHO has defined low fruits and vegetables intake as <5 servings per day [32]. However, using this cut off, almost all the study subjects were under the low intake criteria, therefore, we used the sample median, and classified ≤2 servings of fruits or vegetables daily, as low intake. Those involved in any significant physical activity were classified as active and with >30 minutes of work–, leisure–, or commute–related physical activity were classified as moderately active. Hypertension was diagnosed when systolic blood pressure was ≥140 mm Hg and/or diastolic ≥90 mm Hg or a person was a known hypertensive. Overweight was defined as body mass index ≥25 kg/m^2^ and obesity defined by body mass index ≥30 kg/m^2^. Truncal obesity was diagnosed when waist–hip ratio was >0.9 in men and >0.8 in women or waist circumference was >90 cm in men and >80 cm in women according to the international harmonised guidelines [33]. Dyslipidemia was defined by the presence of high total cholesterol (≥5.2 mmol/L), high LDL cholesterol (≥3.4 mmol/L), low HDL cholesterol (<1.0 mmol/L in men and <1.3 mmol/L in women) or high triglycerides (≥1.7 mmol/L), or if the individual was on treatment with cholesterol–lowering drugs according to US National Cholesterol Education Program [34]. Diabetes was diagnosed on the basis of either history of known diabetes on treatment or fasting glucose ≥7 mmol/L, similar definition was used in our previous report [35]. The diagnosis of the metabolic syndrome was based on the harmonized Asian criteria [33], and was diagnosed when any three were present out of the following five– waist size >90 cm men, >80 cm women; BP systolic ≥130 and/or diastolic ≥85 mm Hg; fasting triglycerides ≥1.7 mmol/L; HDL cholesterol <1.0 mmol/L men, <1.3 mmol/L women; and fasting blood glucose >5.5 mmol/L or known diabetes.

### Statistical analyses

All the case–data were entered into a SPSS database (Version 10.0, SPSS Inc, Chicago). More than 90% data for various variables were available and in more than 85% subjects the data for all the variables were available. For risk factors, the prevalence rates (%) and 95% confidence intervals (CI) for men and women are reported separately. Age–adjustment was performed using direct method with 2001 Indian census population as standard. Prevalence of risk factors in various geographical and social development index groups is reported as percent with 95% CI. Intergroup comparisons have been performed using χ^2^ test. For geographic differences in risk factors we determined differences in prevalence rates in cities in eastern region (where low prevalence of many risk factors was observed) with cities in northern, western and southern regions using χ^2^ test. The χ^2^ test was also performed to identify significance of difference in prevalence of various risk factors in low vs medium and high social index development cities. Trends in risk factors in low, medium and high social development index groups have been examined using least–squares regression and R^2^ values calculated. P values of <0.05 are considered significant.

## RESULTS

The study was performed at 11 cities in India (**Figure 1**). 6198 subjects (men 3426, women 2772) of the targeted 9900 subjects were evaluated (response 62%). Recruitment at individual sites, as proportion of total recruited, was Ahmadabad 490 (7.9%), Bikaner 499 (8.1%), Belgaum 50 (0.8%), Chandigarh 502 (8.1%), Dibrugarh 500 (8.1%), Jaipur 1324 (21.4%), Jammu 320 (5.2%), Lucknow 835 (13.5%), Madurai 923 (14.9%), Nagpur 264 (4.3%) and Patna 491 (7.9%). Data for social and demographic characteristics in men and women have been reported earlier [27]. Men were slightly older than women and there was no significant difference across various age–groups. Low educational status (<10 years of formal education) was more present among women (47.6%) as compared to men (22.3%) and majority of subjects belonged to middle socioeconomic status. More than half of all men and women lived in joint families and 85.6% were married.

Prevalence of various cardiovascular risk factors (age–adjusted prevalence, 95% CI) in the whole group and in men and women are reported in the **Table 3**. There is low prevalence of smoking, smokeless tobacco use as well as alcohol intake in men and women while prevalence of low fruit and vegetable intake, high visible fat intake and sedentary lifestyle is moderate to high. Prevalence of biophysical and biochemical risk factors is also moderate to high.

**Table 3:** Age–adjusted prevalence of lifestyle and cardiometabolic and lifestyle risk factors in men and women*

	Total	Men	Women
**Variables**	**N = 6198**	**N = 3426**	**N = 2772**
Sedentary lifestyle (<moderate physical activity)	42.1 (40.9–43.3)	38.8 (37.2–40.4)	46.1 (44.2–48.0)
Visible fat intake:			
20–40 g/d	35.8 (34.6–37.0)	36.6 (35.0–38.2)	34.7 (32.9–36.5)
>40 g/d	14.1 (13.2–15.0)	14.6 (13.4–15.8)	13.5 (27.2–30.6)
Fruits and vegetables intake (≤2 servings/d)	26.9 (25.8–28.0)	25.3 (23.8–26.8)	28.9 (27.2–30.6)
Smoking:			
Current smokers	6.9 (6.3–7.5)	12.0 (10.9–13.1)	0.5 (0.2–0.7)
Ex–smokers	3.2 (2.8–3.6)	5.1 (4.4–5.8)	0.9 (0.5–1.3)
Smokeless tobacco use	9.8 (9.1–10.5)	12.7 (11.6–13.8)	6.3 (5.3–7.2)
Alcohol consumption:			
<7 drinks/week	7.6 (6.9–8.3)	12.5 (11.4–13.6)	1.5 (1.1–2.0)
≥7 drinks/week	0.3 (0.2–0.4)	0.6 (0.3–0.6)	–
Obesity:			
BMI ≥25 kg/m^2^	42.9 (41.7–44.1)	41.1 (39.4–42.7)	45.2 (43.3–47.1)
BMI ≥30 kg/m^2^	11.6 (10.8–12.4)	8.3 (7.4–9.2)	15.8 (14.4–17.2)
Truncal obesity:			
Waist >90/>80 cm, men/women	45.5 (44.3–46.7)	35.7 (34.1–37.3)	57.5 (55.7–59.0)
WHR >0.9/>0.8, men/women	75.7 (74.7–76.8)	69.0 (67.5–70.6)	83.8 (82.9–85.2)
Hypertension	31.6 (30.4–32.8)	32.5, 30.9–34.1)	30.4 (28.7–32.1)
High total cholesterol (≥5.2 mmol/L)	25.0 (23.9–26.9)	24.8, 23.3–26.3)	25.3 (23.7–26.9)
High triglycerides (≥1.7 mmol/L)	36.9 (35.7–38.1)	41.2, 39.5–42.9)	31.5 (29.8–33.2)
Low HDL cholesterol (men <1.0/ mmol/L woman <1.3 mmol/L)	42.5 (41.3–43.7)	34.1, 32.5–35.7)	53.0 (51.1–54.9)
Diabetes (known or fasting glucose ≥7.0 mmol/L)	15.7 (14.8–16.6)	16.7, 15.5–17.9)	14.7 (13.4–16.0)
Metabolic syndrome	35.7 (34.5–36.9)	32.2 (30.6–33.8)	40.4 (38.6–42.2)

BMI – body mass index, WHR – waist hip ratio, HDL – high density lipoprotein

*Prevalence in percent and 95% confidence intervals in parentheses.

We grouped the cities into northern (Jammu, Chandigarh, Bikaner), western (Ahmadabad, Jaipur), eastern (Lucknow, Patna, Dibrugarh) and southern (Madurai, Belgaum, Nagpur) (**Table 2**). Age–adjusted prevalence of various cardiovascular risk factors in men and women at different geographical locations are shown in **Table 4**. We compared prevalence of lifestyle and cardiometabolic risk factors in eastern region cities with others. This shows that among eastern regional urban participants there is lower prevalence of overweight, hypertension, hypercholesterolemia, diabetes and metabolic syndrome. Participants from northern and southern regions have the highest prevalence of hypertension, hypercholesterolemia and low HDL cholesterol. Prevalence of diabetes and metabolic syndrome is highest among participants in the northern and southern regions. Compared with the national average (from **Table 3**), there is a trend towards greater prevalence of smoking in eastern, truncal obesity in northern, hypertension in northern and eastern, hypercholesterolemia in northern, diabetes in northern and southern and metabolic syndrome in northern and southern Indian cities. However, these differences are not statistically significant.

**Table 4:** Age–adjusted prevalence of various cardiovascular risk factors at cities in eastern, northern, western and southern regions*

	Men				Women			
	**Eastern**	**Northern**	**Western**	**Southern**	**Eastern**	**Northern**	**Western**	**Southern**
	**N = 1101**	**N = 713**	**N = 1062**	**N = 550**	**N = 725**	**N = 608**	**N = 752**	**N = 687**
Physical activity (≥moderate)	47.0 (44.0–49.9)	22.3 (19.2–25.3)	43.0 (40.0–45.9)	42.9 (38.7–47.0)	32.8 (29.4–36.2)	31.2 (27.5–34.0)	57.8 (54.3–61.3)	57.0 (53.3–60.7)
Eastern vs others†	–	11.3, <0.001	3.5, 0.060	2.5, 0.111	–	0.38, 0.538	93.1, <0.001	83.8, <0.001
Visible fat intake ≥20 g/d	53.0 (50.0–55.9)	53.4 (49.7–57.1)	52.2 (49.2–55.2)	43.1 (38.9–47.4)	54.5 (50.1–58.1)	47.2 (43.2–51.8)	52.2 (48.5–55.6)	38.4 (34.7–42.0)
Eastern vs others†	–	0.03, 0.869	0.13, 0.715	14.5, <0.001		7.02, 0.008	0.82, 0.364	36.5, <0.001
Fruit/vegetable intake ≤2 servings/d	22.5 (20.0–25.0)	23.4 (20.3–26.5)	30.0 (27.2–32.7)	24.3 (20.7–27.9)	16.8 (14.1–19.5)	24.0 (20.6–27.4)	38.7 (35.2–42.2)	35.4 (31.8–39.0)
Eastern vs others†	–	0.20, 0.657	15.8, <0.001	0.70, 0.408	–	10.6, 0.001	87.6, <0.001	63.3, <0.001
Smoking	16.0 (13.8–18.2)	20.7 (17.7–23.7)	16.1 (13.9–18.3)	16.5 (13.4–19.6)	0.7 (0.09–1.3)	2.0 (0.89–3.1)	2.2 (1.1–3.2)	1.1 (0.32–1.9)
Eastern vs others†	–	6.70, 0.009	0.01, 1.000	0.08, 0.771	–	4.34, 0.037	6.24, 0.013	0.87, 0.350
Smokeless tobacco use	22.0 (19.5–24.4)	11.4 (9.1–13.7)	7.5 (5.9–9.1)	5.4 (3.5–7.3)*	21.3 (18.3–24.3)	1.2 (0.33–2.1)	0.4 (–0.05–0.85)	2.4 (1.2–3.5)
Eastern vs others†	–	32.9, <0.001	89.9, <0.001	73.4, <0.001	–	115.7, <0.001	159.8, <0.001	108.9, <0.001
Overweight (BMI ≥25 kg/m^2^)	34.4 (31.3–36.9)	44.7 (41.0–48.3)	47.0 (40–50)	38.7 (34.6–42.8)	27.3 (24.0–30.5)	54.6 (50.6–58.5)	49.9 (46.3–53.5)	50.5 (46.7–54.2)
Eastern vs others†	–	19.8, <0.001	35.9, <0.001	2.83, 0.092	–	102.8, <0.001	79.1, <0.001	80.1, <0.001
High waist circumference (men/women >90/>80 cm)	21.0 (18.6–23.4)	46.5 (42.8–51.2)	47.8 (44.8–50.8)	30.5 (26.6–34.3)	45.5 (41.9–49.1)	69.2 (65.5–72.9)	61.4 (57.9–64.9)	55.0 (51.2–58.7)
Eastern vs others†	–	132.3, <0.001	173.3, <0.001	18.3, <0.001	–	75.7, <0.001	37.6, <0.001	12.7, <0.001
High waist–hip ratio (men/women >0.9/>0.8)	80.2 (77.8–82.5)	63.7 (60.2–67.2)	60.2 (57.2–63.1)	76.9 (73.4–80.4)	94.5 (92.8–96.1)	76.8 (73.4–80.1)	73.5 (70.3–76.6)	90.5 (88.3–92.7)
Eastern vs others†	–	61.7, <0.001	104.2, <0.001	2.54, 0.111	–	88.0, <0.001	119.4, <0.001	7.46, 0.006
Hypertension	22.4 (19.9–24.8)	42.5 (38.8–46.1)	35.5 (32.6–38.4)	34.3 (30.3–38.3)	27.4 (24.1–30.6)	39.6 (35.7–43.5)	25.9 (22.8–29.0)	30.3 (26.8–33.7)
Eastern vs others†	–	81.4, <0.001	44.9, <0.001	26.1, <0.001	–	22.2, <0.001	0.43, 0.509	1.37, 0.241
High cholesterol (≥5.2 mmol/L)	15.2 (13.1–17.3)	30.6 (27.2–34.0)	29.8 (27.0–32.5)	26.5 (22.8–30.2)	24.2 (21.1–27.3)	25.5 (22.0–28.9)	28.3 (25.1–31.5)	23.0 (19.8–26.1)
Eastern vs others†		60.6, <0.001	60.1, <0.001	30.3, <0.001	–	0.26, 0.608	3.12, 0.077	0.32, 0.570
High triglycerides (≥1.7 mmol/L)	45.3 (42.3–48.2)	42.4 (38.8–46.0)	37.0 (34.1–39.9)	39.6 (35.5–43.7)	36.6 (33.1–40.1)	35.1 (31.3–38.9)	22.9 (19.9–25.9)	32.5 (29.0–36.0)
Eastern vs others†		1.50, 0.214	15.8, <0.001	4.81, 0.028	–	0.33, 0.564	32.5, <0.001	2.4, 0.119
Low HDL cholesterol (men/women <1.0/<1.3 mmol/L	39.9 (37.0–42.8)	34.4 (30.9–37.9)	24.2 (21.6–26.8)	41.1 (37.0–45.2)	56.6 (53.0–60.2)	56.5 (52.5–60.4)	36.4 (32.9–39.8)	64.2 (66.6–67.8)
Eastern vs others†		5.82, 0.008	61.5, <0.001	0.66, 0.820	–	0.01, 1.00	60.1, <0.001	8.60, 0.003
Diabetes	10.9 (9.1–12.7)	26.8 (23.5–30.0)	13.3 (11.2–15.3)	22.0 (18.5–25.4)	8.5 (6.4–10.5)	19.9 (16.7–23.1)	9.7 (7.6–11.8)	20.9 (17.8–23.9)
Eastern vs others†		76.9, <0.001	2.91, 0.089	36.2, <0.001	–	35.9, <0.001	0.59, 0.444	43.6, <0.001
Metabolic syndrome (Harmonized Asian definition)	23.4 (20.9–25.9)*	46.7 (43.0–50.3)	50.8 (47.8–53.8)	33.8 (29.8–37.7)	31.3 (27.9–34.7)	56.4 (52.4–60.3)	30.3 (27.0–33.6)	44.5 (40.8–48.2)
Eastern vs others†		106.7, <0.001	30.0, <0.001	20.1, <0.001	–	85.1, <0.001	0.17, 0.679	26.3, <0.001

BMI – body mass index, HDL – high density lipoprotein

*Prevalence in percent and 95% confidence intervals in parentheses.

†χ^2^–test statistic and p value.

The cities were also classified according to human social development index into tertiles of low, mid and high groups. Prevalence of various lifestyle and cardiometabolic risk factors in different social development index groups and significance of differences in their prevalence in low vs medium and high social development cities is shown in **Table 5**. Prevalence of smokeless tobacco use, high visible fat intake and low fruits and vegetables intake is greater in participants from low social development index cities as compared to medium and high development cities while low physical activity is more among women in high index cities. Prevalence of cardiometabolic risk factors (overweight/obesity, high waist circumference in men, hypertension, high total cholesterol, diabetes and metabolic syndrome) is significantly greater among participants from cities with medium and high social development index. Prevalence of low HDL cholesterol is more in low social development index cities.

**Table 5:** Age–adjusted prevalence (%) and 95% confidence intervals of cardiovascular risk factors in low, mid and high social development index cities

Variable	Men			Women		
	**Low SDI (n = 1210)**	**Mid SDI (n = 1382)**	**High SDI (n = 834)**	**Low SDI (n = 615)**	**Mid SDI (n = 1252)**	**High SDI (n = 905)**
Physical activity (≥moderate)	37.6 (34.9–40.3)	39.3 (36.7–41.9)	39.8 (36.5–43.1)	37.9 (34.1–41.7)	44.7 (41.9–47.4)	53.7 (50.4)
Low SDI vs others†	–	0.84, 0.358	1.00, 0.314	–	7.90, 0.005	36.7, <0.001
Visible fat intake ≥20 g/d	62.1 (59.4–64.8)	51.7 (49.1–54.3)	38.6 (35.3–41.9)	62.4 (58.6–66.2)	49.5 (46.7–52.3)	37.3 (34.1–40.4)
Low SDI vs others†	–	28.7, <0.001	109.7, <0.001	–	27.6, <0.001	92.4, <0.001
Fruit intake ≤2 servings/d	27.2 (24.7–29.7)	25.7 (23.4–28.0)	21.9 (19.1–24.7)	31.2 (27.5–34.8)	26.2 (23.7–28.6)	31.1 (28.1–34.1)
Low SDI vs others†	–	0.68, 0.410	7.22, 0.007	–	5.21, 0.023	0.01, 0.998
Smoking	17.6 (15.4–19.7)	17.4 (15.4–19.4)	15.7 (13.2–18.2)	0.8 (0.1–1.5)	2.1 (1.3–2.9)	1.0 (0.35–1.65)
Low SDI vs others†	–	0.012, 0.999	1.27, 0.262	–	4.42, 0.035	0.13, 0.716
Other tobacco use	14.6 (12.6–16.6)	16.2 (14.2–18.1)	4.0 (2.7–5.3)	5.0 (3.3–6.7)	10.7 (9.0–12.4)	0.8 (0.22–1.38)
Low SDI vs others†	–	1.22, 0.267	59.4, <0.001	–	16.4, <0.001	21.6, <0.001
Overweight (BMI ≥25 kg/m^2^)	33.9 (31.2–3.66)	47.2 (44.6–49.8)	41.3 (37.9–44.6)	34.3 (30.5–38.0)	44.0 (41.2–46.7)	54.1 (50.8–57.3)
Low SDI vs others†	–	47.6, <0.001	11.9, <0.001	–	16.7, <0.001	57.9, <0.001
High waist circumference (men/women >90/>80 cm)	31.5 (28.9–34.1)	40.7 (38.1–43.3)	33.6 (30.4–36.8)	57.4 (53.5–61.3)	57.0 (54.2–59.7)	58.3 (55.1–61.5)
Low SDI vs others†	–	23.8, <0.001	0.98, 0.321	–	0.02, 0.879	0.13, 0.714
High waist–hip ratio (men/women >0.9/>0.8)	75.2 (72.8–77.6)	60.2 (57.6–62.8)	74.7 (71.7–77.6)	82.1 (79.1–85.1)	80.4 (78.2–82.6)	89.7 (87.7–91.7)
Low SDI vs others†	–	65.3, <0.001	0.07, 0.795	–	0.76, 0.384	18.3, <0.001
Hypertension	27.3 (24.8–29.8)	35.9 (33.4–38.4)	34.4 (31.2–37.6)	28.4 (24.8–31.9)	30.1 (27.5–32.6)	32.0 (28.9–35.0)
Low SDI vs others†	–	21.6, <0.001	11.6, 0.001	–	0.54, 0.461	2.3, 0.124
High cholesterol (≥5.2 mmol/L)	20.0 (17.7–22.2)	26.9 (24.5–29.2)	28.6 (25.5–31.7)	23.4 (20.0–26.7)	26.8 (24.3–29.2)	24.3 (21.5–27.1)
Low SDI vs others†	–	16.5, <0.001	17.5, <0.001	–	2.51, 0.111	0.25, 0.617
High triglycerides (≥1.7 mmol/L)	42.7 (39.9–45.5)	39.2 (36.6–41.8)	42.2 (38.8–45.5)	31.2 (27.5–34.8)	30.8 (28.2–33.6)	32.8 (29.7–35.8)
Low SDI vs others*†	–	3.29, 0.070	0.05, 0.815	–	0.03, 0.864	0.43, 0.512
Low HDL cholesterol (men/women <1.0/<1.3 mmol/L)	41.6 (38.8–44.4)	25.2 (22.9–27.5)	37.9 (34.6–41.2)	57.0 (53.1–60.9)	44.8 (42.0–47.5)	61.5 (58.3–64.7)
Low SDI vs others†	–	79.3, <0.001	2.91, 0.088	–	24.8, <0.001	3.05, 0.081
Diabetes	13.8 (11.8–15.7)	14.1 (12.3–15.9)	25.3 (22.3–28.2)	10.9 (8.4–13.3)	11.0 (9.3–12.7)	21.5 (18.8–24.2)
Low SDI vs others†	–	0.05, 0.821	43.3, <0.001	–	0.01, 1.00	29.1, <0.001
Metabolic syndrome (Harmonized Asian definition)	29.6 (27.0–32.2)	29.6 (27.2–32.0)	40.2 (36.8–43.5)	13.8 (32.6–40.3)	36.0 (33.3–38.7)	47.9 (44.6–51.1)
Low SDI vs others†	–	0.00, 1.00	25.2, <0.001	–	229.5, <0.001	343.4, <0.001

SDI – social development index, BMI – body mass index, LDL – low density lipoprotein, HDL – high density lipoprotein

*Prevalence in percent and 95% confidence intervals in parentheses.

†χ^2^–test statistic and p value.

**Figure 2:**
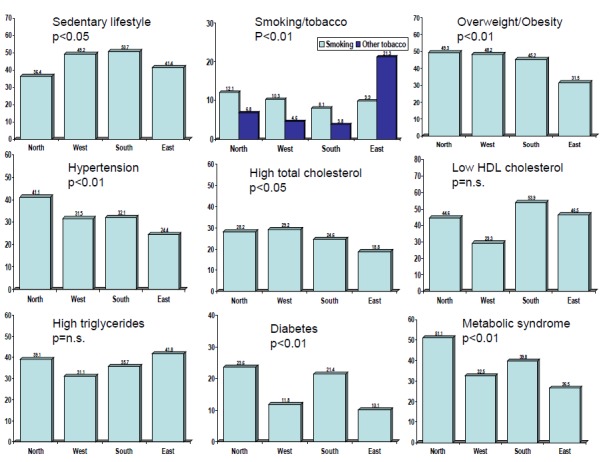
Prevalence of various cardiometabolic risk factors in different geographic regions of India (age– and sex–adjusted). Overweight/obesity, hypertension, high total cholesterol, diabetes and metabolic syndrome is lowest in the cities of eastern region while tobacco use is the highest (p values in **Table 4**).

**Figure 3:**
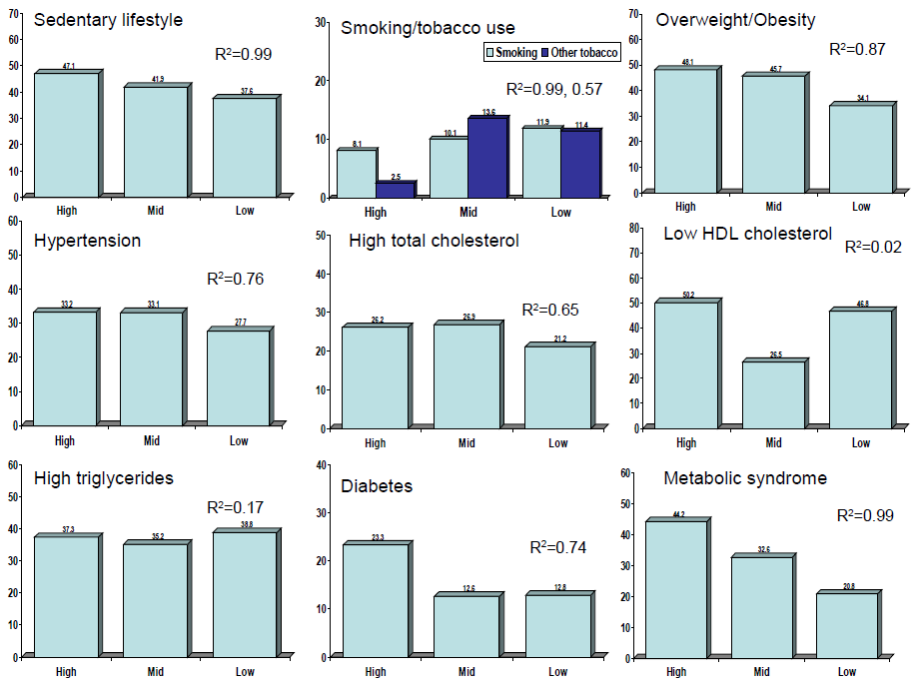
Prevalence of cardiometabolic risk factors in low, mid and high human social development index cities and trends (Least squares regression R^2^).

Age and sex–adjusted prevalence of various cardiometabolic risk factors in subjects in different geographic locations and social development index cities are shown in **Figures 2 **and** 3**, respectively. For geographic locations, there are no significant linear trends in risk factor prevalence among participants from different geographic regions (**Figure 2**). **Figure 3** shows that there are significant trends (R^2^ values) in sedentary lifestyle (0.99), overweight/obesity (0.87), abdominal obesity (0.88), diabetes (0.74) and metabolic syndrome (0.99) in low vs medium and high social development index groups, while the prevalence of smoking (0.99) shows opposite trend (**Figure 3**).

## DISCUSSION

The study shows a high prevalence of cardiometabolic risk factors (hypertension, hypercholesterolemia, hypertriglyceridemia, low HDL cholesterol, diabetes and metabolic syndrome and obesity and abdominal obesity) in Asian Indian urban middle class subjects. These prevalence rates are greater than contemporary regional and national population based studies from different parts in the country [36-44] (**Table 6**). This study shows that metabolic cardiovascular risk factors are lower among participants from cities in eastern India. Study pafrticipants living in cities with lower human social development index have lower prevalence of these risk factors.

**Table 6:** Prevalence (%) of cardiometabolic risk factors in recent multi–centric Indian studies

Study (Year)	Sample size	Overweight/ Obesity	Hypertension	High cholesterol	Low HDL cholesterol	Diabetes	Metabolic syndrome
National Urban Diabetes Survey (2001) [37]	11 216	30.8	–	–	–	12.1	–
National Family Health Survey–3 (2005–6) [36]	198 754	12.6	–	–	–	–	–
Prevalence of Diabetes in India Study: Urban (2004) [38]	21 516	–	–	–	–	4.6	–
Prevalence of Diabetes in India Study: Rural (2004) [38]	19 754	–	–	–	–	1.9	–
Indian Industrial Population Surveillance Study (2006) [40]	10 442	31.5	27.3	–	–	9.7	28.6
Integrated Disease Surveillance Project: Urban (2009) [41]	18 552	24.1	19.9	–	–	–	–
Integrated Disease Surveillance Project: Rural (2009) [41]	19 481	9.7	14.3	–	–	–	–
India Migration Study: Rural (2010) [42]	1983	23.2	20.7	24.5	48.5	5.6	–
India Health Study (2011) [43]	3814	50.3	30.0	–	–	12.3	–
Indian Diabetes Study: Urban/Rural, 4 states (2011) [39]	13 055	–	–	–	–	5.3–13.6	–
Indian Women Health Study: Rural (2011) [44]	2616	22.5	31.5	13.5	–	4.3	–
Indian Women Health Study: Urban (2011) [44]	2008	44.6	48.2	27.7	–	15.1	–
**Present study**	**6198**	**42.9**	**31.6**	**25.0**	**42.5**	**15.7**	**35.7**

In India there have been limited nationwide or multisite studies that used uniform methodologies to assess multiple cardiovascular risk factors as done in the present study (**Table 6**). The National Family Health Survey, which is the largest health survey in the country, reported prevalence of smoking and obesity in urban and rural populations [36]. No other cardiometabolic risk factors were evaluated. The third cycle of this study in years 2005–2006 reported low prevalence of overweight/obesity and moderate prevalence of smoking in Indian urban and rural populations [36]. The multisite NUDS (National Urban Diabetes Survey) [37], PODIS (Prevalence of Diabetes in India Study) [38] and INDIAB (Indian Diabetes) [39] studies were focused on diabetes prevalence. Only limited studies have evaluated multiple cardiovascular risk factors in two or more locations in India using similar methodology [40-44]. The multisite Indian Industrial Population Surveillance Study [40] reported prevalence of cardiovascular risk factors among industrial workers at eight sites in the country and reported lower prevalence of obesity, hypertension, diabetes and metabolic syndrome as compared to the present study (**Table 6**). Indian Council of Medical Research Integrated Disease Surveillance Project [41] in 9 states of the country reported lower prevalence of hypertension in various rural and urban locations in the country as compared to the present study. The India Migration Study reported high prevalence of various risk factors in rural kin of industrial workers [42]. The multisite India Health Study in Mumbai, Delhi and Trivandrum focussed on diet and reported prevalence of overweight and diabetes similar to the present study [43]. Indian Women Health Study involving middle–aged women in 4 urban and 5 rural sites in India reported prevalence of cardiovascular risk factors in urban lower middle class women which were similar to the present study [44]. These studies did not comment on geographical differences. Our study is larger and more diverse than all these previous studies and shows high prevalence of multiple cardiometabolic risk factors in the middle–class Indian urban population. Although the finding of high prevalence of cardiovascular risk factors in Indian urban populations is not unique and has been reported earlier [7], the present study shows that prevalence of cardiometabolic risk factors such as metabolic syndrome, diabetes and atherogenic dyslipidemia (borderline high LDL cholesterol, high triglycerides and low HDL cholesterol) is particularly high among the urban middle–class. This finding is all the more important because large segments of Indian society are entering the middle–class and it has been predicted that within the next 30 years more than 70% of the population (more than a billion) shall be urban and most would belong to this segment of the society [20]. The INTERHEART study reported that metabolic risk factors such as atherogenic dyslipidemia (abnormal apolipoprotein A/apolipoprotein B ratio), truncal obesity, hypertension and diabetes are most important cardiovascular risk factors in South Asians [45]. Our study shows that prevalence of cardiometabolic risk factors is high in urban middle class Indian populations and predicts an impending cardiovascular epidemic in the country unless measures for risk factor control are adopted. Middle class is one of the fastest growing segment of the Indian society and is already more than 400 million subjects strong (30% of total population) [21]. Cardiovascular disease epidemic in such a large population segment would translate into heavy social as well as economic burden on the society [46]. We did not study rural locations, which currently include >60% of the Indian population, and the urban slums. This is a major study limitation and our data are, therefore, not valid for the entire country and only represent the middle class (urban and possibly also the rural middle class).

Lower prevalence of cardiometabolic risk factors and greater prevalence of smoking and smokeless tobacco use in cities of eastern region of the country is an important finding (**Table 4**). This is similar to NFHS studies which reported greater prevalence of smoking and tobacco use (40% men) and lower prevalence of obesity (15%) in this region [12,13]. This geographic region also has a lower human and social development [23,25]. In the present study we have shown that human social development index, which is a composite of six social and economic factors, is an important determinant of cardiovascular risks. The study shows that apparently similar middle class locations in different cities vary in cardiovascular risk according to social and economic development of the cities. The least developed cities (Patna, Lucknow, Bikaner, **Figure 1**) which have the lowest human and social development indices (**Table 2**) have the lowest prevalence of risk factors. These cities are situated in less developed Indian states where problems of communicable diseases and maternal and child health issues are more important [47]. It is likely that once these cities progress to better social and human development indices (better education, income and occupation), cardiovascular risk factors would increase. This finding is in contrast to high income countries, where cardiovascular risk factors are more in low socioeconomic locations, neighbourhoods and states [48,49]. In many low and lower–middle income countries of Asia, Africa and South America, studies have reported findings similar to our study [1,50]. The present study, thus, suggests that populations in India have not achieved the risk factor transition associated with social and economic development where cardiovascular risk factors are greater in low socioeconomic subjects and locations as observed in Europe and North America [1,5]. The human social development index that we used is focussed on macrolevel health and social indicators which, although are indicators of economic and social deprivation (poverty), may not be directly involved in cardiovascular risks. We did not study many other social determinants of cardiovascular health [5] which are equally important risk factors.

### Strengths and limitations

The present study, thus, provides a new insight into the cardiovascular disease epidemic in India. We have highlighted the high prevalence of cardiometabolic risk factors in urban locations in the country. This study also shows importance of poverty decline (social development) as driver of cardiovascular risk. Other strengths of the study include inclusion of almost all the regions of the country; evaluation of risk factors in urban locations that are known to have high cardiovascular disease incidence and prevalence, and use of uniform methodology and measurements, especially biochemical measurements.

Limitations of the study include non–inclusion of large Indian states such as Andhra Pradesh and Madhya Pradesh, but inclusion of locations in other large states such as Uttar Pradesh, Bihar, Rajasthan and Maharashtra is unique to this study. Second, sampling confined to urban locations in middle–level cities could be criticised for selection bias but such urban locations now represent the heart of India [51] and are fertile ground for cardiovascular epidemic. Moreover, rapidly increasing urbanization in the country shall lead to more than 60% of the 1.5 billion subjects living in similar locations within the next 30 years [20,21,51] and there is a need to create more healthy cities focused on social, biological and built environment [18]. Third, the sample size at individual site is too small to identify differences in risk factors. We, therefore, combined cities of a particular geographic region or with similar social development index to determine regional difference in prevalence of various cardiovascular risk factors. Fourthly, sampling confined to middle–class locations in urban areas may not be representative of the city where almost 30% subjects live in slums or in India where more than 65% live in rural locations [47]. However, as mentioned above, the study was focused on middle–level cities and middle–class locations where more than 300 million Indians live [20]. Fifthly, the definition of middle class could vary from state to state and this is a study imitation. We used the criteria adopted by the state governments to classify middle class locations as mentioned earlier. This could lead to non–uniform characterisation of the middle class in the cities. However, as observed in **Table 2**, the sociodemographic pattern of cities in almost uniform. Moreover, we grouped the cities into tertiles of high, middle and low social development index which has resulted in grouping of similar cities with better representativeness. Sixthly, selection of cities, locations and participants could be criticised as biased due to convenience sampling. The best epidemiological approach would have been to divide the cities in the whole country depending on high, middle and low socioeconomic development and randomly select a few, randomly select middle–class locations in individual cities and then randomly select specific areas within the locations and then assess the risk factors in these locations based on either random enumeration of households or a consecutive sampling as done in the present study. We did not randomise the whole cities into strata based on locations and amenities but chose middle class locations, as classified by government records, and then performed the consecutive household survey or simple cluster sampling at all the 11 locations. This method has been recommended by the WHO as alternate strategy to stratified random sampling.^26^ Seventhly, the study has low response rates (62%) which is an important limitation. However, the response rate is similar to many previous studies from India.^7^ It has been opined that response rate of >60% be considered as threshold of acceptability and has face validity as a measure of survey quality [52]. Empirical assessments over the past decade have concluded that the response rate of a survey may not be as strongly associated with the quality of representativeness of the survey as is generally believed. We do not have characteristics of non–responders but neighbourhoods were similar to subjects in the present study. And finally, we have linked individual level outcomes with macro–level predictors (social development index) in the present study and this may be considered a study limitation. However, we believe that study of macro–level predictors of cardiovascular health is important because only limited studies exist from low income countries that have evaluated such associations and also because these determinants are more amenable to social engineering [18].

In conclusion, this multisite cardiovascular risk factor study in India shows that as cities move away from economic and social deprivation there is greater prevalence of obesity, metabolic syndrome, diabetes and dyslipidemias. Behavioural risk factors such as smoking, smokeless tobacco use, low fruits and vegetables intake and high visible fat intake are higher in low social development cities. This indicates that Indian urban populations are in different stages of epidemiological and chronic disease transition– low social development cities have greater prevalence of behavioural risk factors while more developed cities have greater cardiometabolic risk factors. This mixed picture of risk factor transition suggests that the cardiovascular epidemic is still evolving in India, similar to many lower–middle income countries of Europe, Asia, South America and Africa [53]. This study, thus, shows that decline in cardiovascular risks that occurs with social and human development has not yet occurred in India. Studies in developed countries have reported that socioeconomic tansition (captured by social development index in the present study) is inversely associated with cardiometabolic risks and greater social and human development index is associated with lower risks [4,5,53]. Our study shows that in India the regional variations in cardiovascular risk factors related to geography (greater prevalence in eastern India) is related to lower social and human development of this region. This study, therefore, suggests that in India there is need to focus on macrolevel social determinants of cardiovascular risks to control the cardiovascular epidemic. These findings are all the more important in context of WHO 25x25 initiative, 25% reduction in mortality from noncommunicable diseases by the year 2025 [50]. National health policy of India, and many low and low–middle income countries, should be directed to not only improving macrolevel societal factors– amelioration of poverty, inequity, illiteracy, unemployment and undernutrition– that impact health, but also promotion of cardiovascular health with standard public health approaches [1,50]. The most cost–effective approach to cardiovascular disease control are implementation of policy measures highlighted by WHO [50] to attain the nine global noncommunicable disease targets, all of which are focussed on cardiovascular diseases.
